# Robotic Total Knee Arthroplasty: An Update

**DOI:** 10.3390/jpm14060589

**Published:** 2024-05-30

**Authors:** Gennaro Pipino, Alessio Giai Via, Marco Ratano, Marco Spoliti, Riccardo Maria Lanzetti, Francesco Oliva

**Affiliations:** 1 Department of Orthopedic Surgery and Traumatology Villa Erbosa Hospital, Gruppo San Donato, 40129 Bologna, Italy; gennaro.pipino@grupposandonato.it; 2 San Raffaele University, 20132 Milan, Italy; 3 Department of Orthopedic Surgery and Traumatology, San Camillo-Forlanini Hospital, 00152 Rome, Italy; mspoliti@scamilloforlanioni.rm.it (M.S.); rm.lanzetti@scamilloforlanini.rm.it (R.M.L.); 4 Unit of Orthopaedics, Department of Life, Health and Environmental Sciences, University of L’Aquila, 67100 L’Aquila, Italy; marco.ratano@graduate.univaq.it; 5 Full Professor Department of Human Sciences and Promotion of the Quality of Life, San Raffaele Roma Open University, 00166 Rome, Italy; francesco.oliva@uniroma5.it

**Keywords:** total knee arthroplasty, robotic total knee arthroplasty, computer assisted surgery, arthrosis

## Abstract

Total knee arthroplasty (TKA) is a gold standard surgical procedure to improve pain and restore function in patients affected by moderate-to-severe severe gonarthrosis refractory to conservative treatments. Indeed, millions of these procedures are conducted yearly worldwide, with their number expected to increase in an ageing and more demanding population. Despite the progress that has been made in optimizing surgical techniques, prosthetic designs, and durability, up to 20% of patients are dissatisfied by the procedure or still report knee pain. From this perspective, the introduction of robotic TKA (R-TKA) in the late 1990s represented a valuable instrument in performing more accurate bone cuts and improving clinical outcomes. On the other hand, prolonged operative time, increased complications, and high costs of the devices slow down the diffusion of R-TKA. The advent of newer technological devices, including those using navigation systems, has made robotic surgery in the operatory room more common since the last decade. At present, many different robots are available, representing promising solutions to avoid persistent knee pain after TKA. We hereby describe their functionality, analyze potential benefits, and hint at future perspectives in this promising field.

## 1. Introduction

Despite progressive improvements in prosthetic designs and alignment techniques, up to 20% of patients still report knee pain or are unsatisfied with conventional total knee arthroplasty (C-TKA) [1]. Since the first computer-assisted navigation (C.A.N.) knee replacement surgery in 1997, the use of technology in total knee arthroplasty (TKA) has significantly increased with the aim to improve component placement and lower limb alignment. C.A.N. technology employs either infrared (IR) or electromagnetic (EM) signals to provide real-time feedback during surgery, assisting in instrument positioning and bone resection. However, both IR and EM methods have some limitations, such as visibility issues and interference from metal objects. Despite these technological advancements, a long-term study showed that only 85% of patients were satisfied, and no significant improvements in implant survival rates have been demonstrated at 10-year follow-up compared to C-TKA [2]. With the advent of robotics, common manual inconveniences including incorrect planning, movement of cutting guides, inaccurate bone cuts, restricted range of movement (ROM), inadequate gap balancing, and, finally, incorrect implant size and implant malpositioning could be overcome, reducing persistent knee pain after replacement.

Many studies showed that neutral alignment may improve the functionality of the knee and implant survival during a long-term follow-up, but greater than 3° malalignment is estimated to occur in up to 30% of patients who undergo C-TKA [3]. Therefore, robotic-assisted TKA (R-TKA) was introduced to improve the implant positioning and lower limb alignment, and it could potentially improve patient functional outcomes and survivorship of implants. The term robotics, firstly derived from the Czech term *robota*, i.e., hard job, indicates machine devices that, under a surgeon’s control, facilitate a task in the operatory theatre. Many different robotic devices are available for clinical practice, depending on the degree of freedom of action. They can be classified into active, which perform their task autonomously, semi-active, which provide haptic feedback to avoid potential harms, and passive, which are continuously governed by the operator [4]. Results are conflicting and still unclear. Although several comparative studies showed the improvement of component and limb alignment with R-TKA compared with C-TKA [5,6], no statistical differences have been reported according to clinical outcome scores, aseptic loosening, and overall survivorship [7,8].

The use of robotic technology in surgery also involves initial and ongoing costs, including installation, maintenance, preoperative imaging, extended operation times during the learning phase, training for surgical teams, software updates, service agreements, and consumables. The price of purchasing a robotic device typically ranges from USD 600,000 to USD 1.5 million, depending on its specifications, support, upgrades, and included application systems. However, these costs may be justified by the benefits of robotic total knee arthroplasty (R-TKA), such as reduced opioid usage, decreased need for inpatient physiotherapy, shorter hospital stays, lower readmission rates, and fewer transfers to rehabilitation or skilled nursing facilities compared to conventional TKA techniques [9].

The aim of this study is to summarize the different robotic devices actually available for clinical practice, describing their functionality and analyzing the potential benefits, review the outcome of R-TKA, and outline future perspectives in this field.

## 2. Materials and Methods

This work represents a descriptive review on computer-assisted TKA.

In September 2023, a search of 4 databases (PubMed, Web of Science, Google Scholar, and Embase) was performed using the terms “Total knee arthroplasty”, “robotic surgery”, “computer assisted orthopaedic surgery”, “surgical navigation system” and “conventional” as keywords.

Search results were limited to articles written in English and published in the last 10 years. The search with the criteria used provided a total of 266 articles.

Only comparative studies between primary R-TKA and C-TKA investigating radiological and clinical outcomes, survivorship, and complication rates were included (9 studies). Case series, reviews of the literature, and studies not published in English were excluded from our analysis. Studies involving unicompartmental and revision knee arthroplasty were also excluded.

## 3. Type of Robotic Devices

During the late 90s, CASPAR (URS Orto-Maquet, Schwerin, Germany) was one of the first robotic implants adopted for knee arthroplasty [10]. Like several succeeding robots, it used a femoral and tibial tracker pin for calibration and control, detected with an infrared camera system put separately in the operatory room. Afterwards, with an intraoperative CT scan, the desired implant sizes are selected and, with the knee firmly secured, cuts are initiated by the robot.

Meanwhile, two other robotic devices, the ACROBOT (Imperial College of London, UK) and PiGALILEO (Plus Orthopedics Ag, Rotkreuz, Switzerland), entered the market. Both devices were based on preliminary CT acquisition of bone surfaces. The first is a semi-active system that utilizes a robotic arm connected to a saw to perform precise bone cuts (also for UKA) [11], while the second is a passive device helping in jigs positioning and soft tissue balance [12].

All these systems are open source, allowing for the use of different prosthetic designs. Initial results showed increased correspondence with preoperative planning, reduced bone loss, and clinical scores that were noninferior to C-TKA [2]. However, their use in common practice was cramped by a high complication rate including increased blood loss, infections, patellar fractures, and implant dislocations [2]. Furthermore, other issues were preliminary CT acquisition with radiation exposure, the high running costs of these devices, and increased intraoperative time.

These drawbacks did not discourage medical companies which introduced new devices into the market. Currently, the following medical devices are available (Table 1) (Table 2).

Mako (Stryker, Kalamazoo, MI, USA) uses CT-based planning which is then compared to bony landmarks registered intraoperatively. It provides jigs-free cuts with a semi-active saw or burring. This way, dynamic, millimetric bone resections can be performed without rigid fixation or pin insertion. It was the first currently used robot able to perform total hip replacement (THA), TKAs, unicompartmental knee arthroplasty (UKAs) and bi-compartmental prothesis [30]. Radiographic scores and clinical satisfaction do not change between different implant designs [14]. Short-term studies showed promising results in clinical satisfaction [34], no increase in complication rates, and shorter hospital stays [19]. A greater discharge rate was also accompanied by a slight decrease in surgical time, possibly due to the automated decision of the right (thinner) insert [20]. The learning curve is reached in about 18 cases [21].

Omnibot (Corin, Tampa, FL, USA) does not require preoperative imaging, jigs are mounted after performing the tibial cut, and, through a laminar spreader, it was the first robot to provide soft tissue balancing estimates and adjust femoral cuts accordingly. OMNIBOT requires nine cases for the learning curve with increased surgical time of around 20 min [35]. Several TKAs have been conducted demonstrating great implant survival at six-year follow-up [36].

T-Solution One (THINK Surgical Inc Technology, Fremont, CA, USA), derived from Robodoc, is the only open-source system currently available, i.e., it can use implants from different manufactures. It uses a CT scan to identify required resections and, through milling, autonomously prepares tibial and femoral surfaces. Initial outcomes showed great cut accuracy [15] and precise prosthesis alignment [22]. Ten-year follow up, instead, failed to show significant radiological accuracy or clinical improvements, with complication rates like C-TKA [27].

Rosa (Zimmer-Biomet, Warsaw, IN, USA), commercially available since 2018, utilizes conventional X-ray images to obtain a precise 3D estimate of bony landmarks. After deciding component alignment, the robot automatically positions cutting guides and helps the operator with bone resections [37]. It is also accompanied by a sensor device that detects ligament tension and helps in performing soft tissue releases correctly [23]. The learning curve is reached in around nine cases [24]. Results obtained so far show precise alignment [16] and high patient satisfaction [31].

Cori (Smith and Nephew, London, UK, recently introduced after its predecessor Navio, is a lighter, hand-held device able to obtain a virtual image through pre-determined reference points, thus not requiring preoperative images. It assesses ligament balance and provides precise estimates of final soft tissue tension. A semi-active burr mounted to the robot removes the desired bone. It is useful both for UKA and TKA implants by the same producer. In early 2022, Cori has received FDA clearance for cementless TKAs and revision surgery, making it the first robot approved for revision surgery [32]. Studies on Navio showed improved joint line restoration [33] and component alignment than C-TKA [25]. Short-term clinical scores with Navio or Cori are higher compared to C-TKA, but intraoperative time is longer due to milling [38]. The learning curve is reached after around 25–29 cases [17,26]. The use of this device did not correlate with increased complications, including deep vein thrombosis, compared to C-TKA [28].

Velys (DePuy Synthes, Warsaw, IN, USA), commercially available from mid-2021, is a semi-active robotic arm with a saw attached to it, currently used only for TKA. It is a bed-mounted, relatively narrow, image-free device which detects intraoperative bony landmarks and provides final estimates of gap resections and ligament tension, ensuring that the anticipated soft tissue balance is determined before bone resections. Then, the semi-active saw allows for dynamic tibial and femoral cuts. A cadaveric study showed decreased alignment errors with respect to contralateral C-TKA [29]. Preliminary single-center limited data suggest good clinical results and patient satisfaction at less than one-year follow-up [39]. The learning curve is reached after approximately 20 cases, with no changes in torniquet time and pain scores compared to C-TKA [40].

Skywalker (Microport Navibot, Foxborough, MA, USA) obtained FDA clearance in December 2022. It uses preoperatory CT to position jigs and to perform semi-automated osteotomies. Preliminary results in China show good bone resection accuracy [41] and greater component alignment, with no changes in functional scores [42]. Surgical time is increased, while blood loss is unexpectedly decreased [43].

## 4. Results

### 4.1. Range of Movement

Compared with C-TKR, R-TKR resulted in less loss of ROM immediately after surgery and faster recovery of ROM within three months after operation. Cadmon et al. [44] showed that active ROM was significantly greater than 5.1° and 2.9° at one- and three-month follow-up, respectively, in patients operated with R-TKA compared to C-TKA. With respect to preoperative ROM, the improvement was higher in patients with R-TKA compared to C-TKA: 6.9° at 1-month follow-up and 4.9° at 3-month follow-up. Active extension was a little bit lower for R-TKA (0.44°: *p* = 0.029), but the authors reported no significant differences in passive extension between the two groups of patients.

At long-term follow-up, knee ROM is significantly improved in both robotic and conventional TKA compared to preoperative status, but no statistically significant differences have been reported [44,45]. Lee et al. [46] reported on 855 knees (194 robotic group, 391 navigational group, and 270 conventional group) comparing clinical and radiological outcomes during a minimum follow-up period of 10 years. In the study, the survival rate was analyzed using the Kaplan–Meier method based on the survival endpoint. The analysis of ROM, HSS, KSS, and WOMAC scores were used for clinical evaluation.

ROM and all the other outcome scores improved in the three groups but without any significant difference.

### 4.2. Clinical Evaluations

The most common outcome score to evaluate functional and clinical outcomes were the Visual Analogue Score (VAS) for pain, the Hospital for Special Surgery (HSS) score, the Knee Society Score (KSS, pain, and function), and the Western Ontario and McMaster Universities Osteoarthritis Index (WOMAC) scoring system.

The outcome score significantly improved after surgery both for R-TKA and C-TKA compared to preoperative score. Some authors reported better VAS scores after surgery and better outcome scores for patients who received R-TKA at short-term follow-up (1 and 2 months after surgery), although the differences were not statistically significant. However, well-conduced RCTs reported no significant differences in clinical outcomes between R-TKA and C-TKA both at short-term and at more than 10 years follow-up [46,47,48,49,50]. No differences have been reported in terms of residual pain [48,49]

## 5. Radiographic Outcomes

Postoperative alignment parameters have been studied. R-TKA is statistically superior in restoring the tibiofemoral and mechanical axes, but results are heterogeneous. The prevalence of outliers is lower in R-TKA compared to C-TKA when we consider the desirable knee alignment to be within +/− 3° of a neutral mechanical axis (14% vs. 26%), but if we assume a tolerance level of +/− 5° from a neutral mechanical axis, the difference is not statistically significant [5,47,50,51,52]. There is also no difference in rotational alignment of the femoral component from the transepicondylar axis or tibial component rotation. Lee at al. [46] reported that only the proportion of outliers in the hip–knee–ankle (HKA) axis angle was better for R-TKA, but no other significant differences in radiological outcomes have been reported. Long-term follow-up studies reported a peri-implant osteolysis from 5% to 7%, but no significant differences have been reported between R-TKA and C-TKA [47,51].

## 6. Complications

Despite some retrospective studies reporting lower complication rates in patients who received R-TKA, including anemia, deep vein thrombosis, and pulmonary embolism, more recent well-conduced RCTs showed no significant differences in complications, including aseptic loosening, knee stiffness, and postoperative infections [6,47,52,53]. Furthermore, some specific complications related to R-TKA have been suggested, such as pin-site fracture and pin-tract infection, but none of these complications have been reported in recent comparative studies [7].

## 7. Operative Time

Few level-I studies reported the differences in operative time between R-TKA and C-TKA. The range for R-TKA was 74.50 ± 22.08 min (range, 45–140), which was like the opera-tive time of the C-TKA group (71.74 ± 20.63 min, range: 50–160 min, *p* = 0.55) [52].

## 8. Component Survivorship

There are no differences in revision rate for aseptic loosening between R-TKA and C-TKA. The cumulative survival rate, excluding septic revisions, range from 97.4% to 96.6%, and Kaplan–Meier survival analysis showed no statistically significant differences at 10-year follow-up [8,46,47].

## 9. Cost Analysis

Although there are promising results for R-TKA, the cost–benefit ratio is under investigation.

A recent analysis [54] compared robotic-assisted total knee arthroplasty (R-TKA) with conventional total knee arthroplasty (C-TKA) using a Medicare fee-for-service dataset. Authors found that the 90-day episode- of- care (EOC) was significantly lower for the R-TKA group. Factors contributing to these reduced costs included shorter hospital stays, a higher percentage of patients discharged home, decreased readmission rates, and fewer days spent in skilled nursing facilities and receiving home health visits for those utilizing services. Overall, 90-day episode-of-care-costs were USD 2391 less costly for R-TKA (*p* < 0.0001), driven by fewer readmissions (5.2% versus 7.8%) and greater home discharges (56.7% versus 46.7%). However, the study highlights that as diagnosis-related group payments vary by hospital, the ability to determine claims costs versus true hospital facility costs are difficult to elucidate across the overall population. The current literature suggests that the initial capital investment has the potential to be offset by improved PROMs, shorter length of stay, less pain and reduced opioid intake, higher home discharge, and inferior readmission rates at short-term follow-up [54,55,56].

## 10. Discussion

TKA is a reliable and effective procedure to reduce pain and restore function in patients with severe osteoarthritis. However, about 15% to 25% of patients report pain after knee replacement, and in several patients the reason is not clear. The reported incidence of unexplained, painful TKA varies from 4% to 13.1% [13,57,58]. Accuracy of limb alignment, implant position, and soft tissue balance are considered important factors in preserving unexplained, painful TKA, and R-TKA, improving component positioning and limb alignment, is believed to be effective in reducing such pain. Many robotic systems are available for clinical practice, and research interest on R-TKA is on the rise [18]. In 2022, R-TKA was conducted in less than 10% of TKAs in the US, but the number is expected to increase [59]. In recent years, the number of studies about R-TKA increased but results are still contrasting, and more high-quality clinical trials are necessary to properly establish the superiority of R-TKA compared to T-TKA and the impact on implant survivorship.

The potential benefits of R-TKA are the intrinsic accuracy of the machine, which allows for great reproducibility through standardized actions that minimize procedural errors, the lack of intramedullary guides, the decreased manual stress to ligaments, and that precise bone cuts performed by the robot may result in decreased soft tissue injury and decreased inflammatory markers after surgery [59,60]. This may account for increased ROM [61], decreased postoperative pain and hospital stay, and faster rehabilitation [62]. Better knee ROM and functional outcomes at short-term follow-up have been reported by several authors [37,63]. This is probably due to the better component positioning and the introduction of functional alignment. The advent of R-TKA allowed for the placement of implant prosthetic components in a more anatomical and patient-specific position, preserving bone stock and the native ligament envelope [64]. Better ROM has been associated with patient satisfaction and improved knee function, which are early indicators of a successful procedure. As R-TKR is considered to improve ROM at short-term follow-up [44,65], it may be also associated with improved early patient satisfaction compared to C-TKR. However, well-conduced RCTs at longer follow-up (more than 10 years) fail to find any statistically significant differences in term of knee ROM, PROMs, and overall survivorship between R-TKA and C-TKA [8,66].

Several studies compared final alignment to preoperative planning and resulting functional scores. Retrospective studies show superior, or at least noninferior, accuracy in implant positioning and clinical satisfaction. However, the clinical importance of these findings and their impact on patient outcomes and satisfaction remains uncertain. Yang et al. [52] recently reported that R-TKA improved lower-limb coronal alignment, sagittal implant position, and early functional recovery in patients with severe varus/valgus deformity of the knee, but they did not find significant advantages for the mild varus/valgus deformity over C-TKA. The restoration of both mechanical and functional alignment is better for R-TKA due to improved resection accuracy, implant alignment, and gap balancing, but these outcomes do not appear to result in better functional outcomes and revision rates at long-term follow-up. A recent review [67] suggests that both neutral (0°–3°) and mild varus (3°–6°) alignments post-TKA result in similar patient outcomes in patients with preoperative varus knees. These findings may also be influenced by potential confounding factors, such as different implant designs, different surgical experience, and non-standardized pre- and postoperative protocols. Therefore, more comprehensive exploration of the role that alignment plays in the success of T-TKA, and long-term clinical trials are needed to investigate the impact of limb alignment on implant survivorship.

Despite optimistic projections, R-TKA is not free of risks. Major complications occurred with first-generation, active devices, and current devices still have a series of downsides which need to be addressed [68]. Among them, femoral and tibial tracker pin positioning used for calibration are at risk of resulting in iatrogenic fractures and may be responsible for local complications, such as pin-tract infections [69]. Conversely, accurate distal femoral pin positioning in the medial sagittal plane does not result in iatrogenic fractures [70]. On the other hand, other authors reported in a retrospective study that R-TKA was associated with a decreased complication rate, including deep vein thrombosis and pulmonary embolism, anemia, and decreased opioid consumption, compared to T-TKA [71]. Further well-designed studies are therefore required to compare the rate and type of complications between the two procedures.

The increased surgical time in robotic surgery is another frequently reported issue. The reason is multifactorial, in part inherent to the robot itself (initial set up, pins insertion and calibration, and milling/cutting process), and partly due to the learning curve. For surgeons new to R-TKA, the learning curve takes about 15 cases to gain confidence and around 30 cases are needed to reach operating times like the conventional technique [72,73,74]. After the learning curve, experienced and high-volume surgeons reported similar surgical time within one year between the two techniques [74]. Notably, involvement of trainees in knee arthroplasty does not lengthen operative time [75], making it useful for learning purposes. Increased procedural time inevitably results in increased blood loss. However, recent studies show noninferior postoperative hemoglobin levels or need of blood transfusion despite longer operative time [46,76].

R-TKA remains costly, with prices ranging from USD 400 thousand to more than USD one million depending on the device and the contract. Average three-month health-related costs may be similar [77] or increased [78] compared to C-TKA, although the supply cost might not be mitigated even with hundreds of cases. Recent studies suggest that the initial capital investment could be paid off thanks to reduced postoperative pain and opioid intake, shorter length of stay, higher home discharge, and inferior readmission rates at short-term follow-up. On the other hand, the cost savings may not be as substantial, and longer-term studies clearly establishing these cost savings are lacking. Therefore, sometimes insurance companies do not authorize the payment for R-TKA surgery.

As technology progresses, we can anticipate the development of more efficient, interactive robotic systems that seamlessly integrate augmented reality (AR). Tsukada et al. [79] conducted studies on the AR-KNEE navigation system, first in a pilot study using 10 femoral bone specimens to verify its accuracy, then in a clinical study on 74 patients to compare AR-TKA with a conventional intramedullary guide [80]. No preoperative CT scan was required, and real-time information was provided on a smartphone, allowing surgeons to visualize desired angles during surgery. Authors reported that differences between angles measured using CT images and those displayed on the smartphone were less than 1°. Additionally, Iacono et al. [81] reported on the AR-Knee navigation system in a clinical pilot study involving five TKA cases. This system allowed surgeons to view tibial and femoral axes on smart glasses without additional preoperative images. The authors reported a coronal error of 1° for both tibial and femoral cuts, and a sagittal error of 1.6° for tibial cuts. Discrepancies between angles measured on standard X-rays and those reported with the AR system were also minimal.

Effectively defining the prospects and applications of robotic TKA poses a considerable challenge. This is due to several reasons: firstly, its evolution is exceptionally rapid, with new designs, software, applications, and updates emerging each year. This places significant pressure on healthcare systems to keep pace with such innovations without external funding. Secondly, these devices currently enjoy strong support following promising short-term results across various machine variants.

The decision to limit the bibliographic research to the most recent literature is because the purpose of this article was to perform a descriptive and not a systematic review of the literature. This is certainly a limitation, but it must be also considered that all the recent literature is strongly influenced by the previous research. Many comparative studies are retrospective, which may lead to both over- and under-reporting of data. Furthermore, retrospective studies may produce potential bias during patient selection as surgeons may decide to perform R-TKA for more complex deformities, which could influence the results of the studies.

## 11. Conclusions

Not all robotic devices are equal. The ideal robot should be hand-held, image-free, semi-active, jigs-free, open-source, and possibly implemented with artificial intelligence to provide preoperative and postoperative decision making to further increase clinical results and patient satisfaction. R-TKA improves resection accuracy, implant alignment, and gap balancing, and might decrease future revision rates. R-TKA seems to also reduce the global healthcare cost for patients undergoing TKA by reducing hospital stays, the need for therapies, and readmission rate, but more studies are required to establish the real cost savings. While robots may still be in the early stages of realizing their full potential, they already exhibit advantages compared to conventional techniques. As technology advances, it is possible to anticipate the development of more streamlined, interactive, and augmented reality-integrated robotic systems. Short-term clinical results are encouraging and certainly noninferior to C-TKA. Long-term results of implant survival and clinical satisfaction are still pending but encouraging, and in the next years will reveal whether R-TKA is a standard of care.

## Figures and Tables

**Table 1:** Comparison of different systems’ properties for the currently used robots in total knee arthroplasty.

	Mako	Omnibot	T-Solution One	Rosa	Cori	Velys	Sky-Walker
Source	Closed	Closed	Open	Closed	Closed	Closed	Closed
Implant	TKA, UKA, PFA	TKA	TKA	TKA	TKA, UKA	TKA	TKA
System	Semi-active	Semi-active	Active	Semi-active	Semi-active	Semi-active	Semi-active
Action	Saw	Jig positioning	Milling	Jig positioning	Milling	Saw	Saw
Fixation Jigs	No	Yes	Yes	Yes	No	No	Yes
Imaging	CT	NO	CT	X-Ray	NO	NO	CT
Soft tissue balance	Yes	Yes	No	Yes	Yes	Yes	No

**Table 2:** Comparison of different results on the currently used robots in total knee arthroplasty. Legend: ↑ increased, ↓ decreased, = equal.

	Mako	Omnibot	T-Solution One	Rosa	Cori	Velys	Sky-Walker
Surgical time	↓ [2] ↑ [13]	↑ [14] ↑ [13]		= [15]	↑ [16] ↑ [13]	= [17]	
Radiographic outcomes	↑ [18]	↑ [18]	↑ [19] ↑ [20] = [21]	↑ [22]	↑ [23] ↑ [24] ↑ [18]	↑ [25]	↑ [26]
Clinical outcomes	↑ [11] ↑ [12]		↑ [20]	↑ [27]	↑ [16]	↑ [25]	= [28] = [29]
Learning curve	18 cases [30]	9 cases [14]		9 cases [15]	25 cases [31] 29 cases [32]	20 cases [17]	
Hospital stay	↓ [12]						
Complication rate	= [12] ↓ [29]	= [18]	= [20]	= [15]	= [33]	= [17]	↓ [29]
Implant surgivorship		99% at 6 years [34]					

## Data Availability

This study did not report any data.
